# Tuberculosis—the Face of Struggles, the Struggles We Face, and the Dreams That Lie Within

**DOI:** 10.3201/eid2403.170128

**Published:** 2018-03

**Authors:** Patrick K. Moonan

**Affiliations:** Centers for Disease Control and Prevention, Atlanta, Georgia, USA

**Keywords:** tuberculosis and other mycobacteria, phthisis, Hippocrates, consumption, India, malnutrition, cachetin-induced, cytokine, World TB Day, art, social conscience, bacillus, bacteria

Tuberculosis disease, or phthisis (ϕθίσις, the Greek word for consumption), was named by the father of allopathic medicine, Hippocrates (c. 460–370 BCE), because the disease appeared to consume the affected person through substantial weight loss and wasting (*1*). Hippocrates warned his students against treating persons in late stages of tuberculosis, because nearly all of their patients would die, which would likely tarnish their reputations as healers (*2*).

Today, >10 million persons become ill with tuberculosis, and 2 million die from the disease each year (*3*). India accounts for the largest number of persons with tuberculosis and tuberculosis-related deaths in the world. In 2016, 1.9 million tuberculosis cases were reported to the Revised National Tuberculosis Programme of India (*3*). An additional 1.2–5.3 million patients were estimated to have received treatment for the disease in the private sector of India but remained unrecognized by the global surveillance system (*4*). Many millions of future case-patients will emerge from the huge reservoir of an estimated 354 million persons currently infected with tuberculosis in India (*5*).

India has a large burden of poverty and malnutrition among both adults and children. Malnutrition has wide-ranging effects on health, including increased susceptibility to infectious diseases such as tuberculosis (*6*). In 2016, malnutrition was the leading risk factor for 14.6% of the total disability-adjusted life-years for all-cause illness and death in India (*7*). More than half of all cases of active tuberculosis among women (55%, 95% CI 27%–76%) and men (54%, 95% CI 26%–75%) in India are attributable to susceptibility caused by malnutrition (*8*).

Having been inspired by the faces behind tuberculosis, the artist Stefan Prakash Eicher (http://www.stefaneicher.com/), born in Maharashtra, India, captures the essence of the term “consumption” through his portrait of an emaciated and wasting man rescued from the streets of New Delhi. In *What Dreams Lie Within* (Figure), dark tones represent muscle atrophy recessed against a bony torso and sunken cheeks to highlight the debilitating cost of advanced stages of tuberculosis, partly due to catabolic losses by a cachetin-induced mechanism (a macrophage-secreted cytokine) (*9*) and malnutrition (*10*). Billowing clouds and a blue sky of hope within his eyes subtly support the struggle of survival found within the intensity of his furrowed brow.

**Figure:**
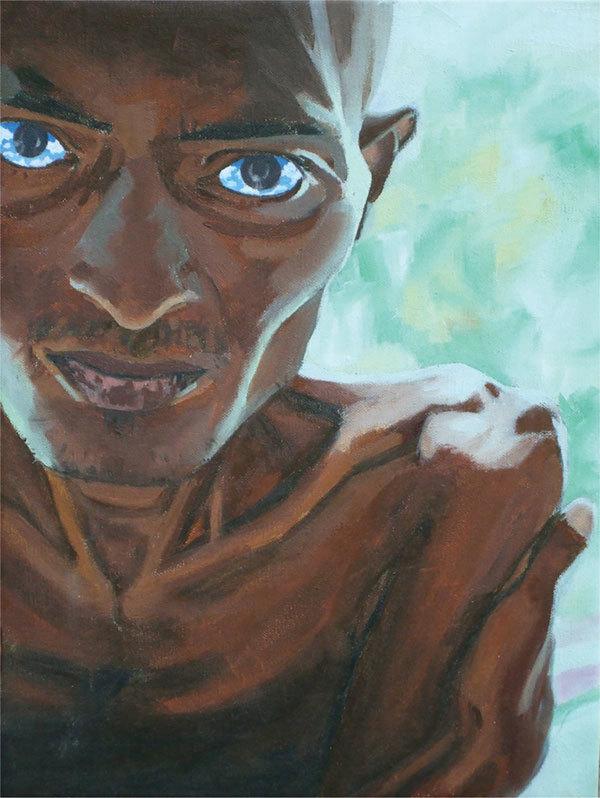
*What Dreams Lie Within* by Stefan Prakash Eicher. Oil on canvas, 2009.

Each year on March 24, we commemorate World TB Day (https://wwwnc.cdc.gov/EID/page/world-tb-day) in honor of the day Robert Koch announced at the University of Berlin Institute of Hygiene that he discovered the cause of consumption, the tuberculosis bacillus. World TB Day is a time to remember the millions of faces of persons who suffer from tuberculosis, to reflect on struggles we face as public health practitioners to end the epidemic, and to find hope in the eyes of the patients we treat and in the dreams that lie within our surviving patients searching for a better tomorrow.
